# Etymologia: Quinine

**DOI:** 10.3201/eid2107.ET2107

**Published:** 2015-07

**Keywords:** etymologia, quinine, malaria, drug, alkaloid, cinchona, bark, fever trees

## Quinine [kwinʹin]

From the Quechua *kina*, “bark,” quinine is an alkaloid of cinchona that has antimalarial properties. In the 1620s, Jesuit missionaries living in Peru learned of the healing powers of the bark of “fever trees” that grew in the high forests of Peru and Bolivia.
